# Use of indocyanine green and the HyperEye system for detecting sentinel lymph nodes in breast cancer within a population of European patients: a pilot study

**DOI:** 10.1186/s12957-016-1060-9

**Published:** 2016-12-01

**Authors:** Oldřich Coufal, Vuk Fait

**Affiliations:** 1 Department of Surgical Oncology, Masaryk Memorial Cancer Institute, Žlutý kopec 7, 656 53 Brno, Czech Republic; 2 Department of Surgical Oncology, Faculty of Medicine, Masaryk University, Kamenice 753/5, 625 00 Brno, Czech Republic

**Keywords:** Breast cancer, Sentinel lymph node biopsy, Indocyanine green, Fluorescence, HyperEye

## Abstract

**Background:**

Certain studies suggest that using indocyanine green (ICG) could be comparable with using radioisotopes (RI) in detecting sentinel lymph nodes (SLNs) in breast cancer. A number of these studies were performed in Asia. The objective of our pilot study was to evaluate within a European population of breast cancer patients the detection rate of SLNs using ICG and the HyperEye system and the concordance in SLNs detected using this method and the standard method involving RI and a gamma probe.

**Methods:**

Ten female patients with early-stage breast cancer (Czech Republic) indicated for partial mastectomy and SLN biopsy were subjected to standard application of RI. Before surgery, ICG was administered periareolarly in the amount of 1 ml of 0.5% solution. Sentinel lymph nodes were first detected perioperatively exclusively using ICG fluorescence and the HyperEye device (Mizuho, Japan). Only after removal of all SLNs found in this way was the standard hand-held gamma probe used to detect RI, and any potential additional SLNs not found with ICG were then extirpated.

**Results:**

In all 10 cases, at least one SLN was successfully detected using ICG. Nevertheless, in five patients, 1–4 additional SLNs were found using the gamma probe. Complete concordance in detecting SLNs therefore occurred in only one half of the cases. Metastases in SLNs were found in a total of two cases. Had we used only ICG for detection, one of these two cases would have been incorrectly evaluated as N0 (ICG false negativity).

**Conclusions:**

The study did not confirm the hypothesis that the use of ICG with the HyperEye system can currently be considered a method fully comparable with using RI and a gamma probe in a population of European patients. Although the detection rate is high, a significantly lower number of SLNs were detected using ICG than using RI (*p* = 0.03). Thus, there would be a higher probability for false negatives to occur in using SLN biopsy. This is caused mainly by the limited permeability of tissues to fluorescent radiation and the difficulty therefore of detecting nodes located deeper beneath the body’s surface.

## Background

Sentinel lymph node biopsy is today a standard surgical procedure used in treating breast cancer and certain other tumours. In practice, the location of a sentinel lymph node (SLN) is determined using a tracer which is usually radioactively marked albumin (radioisotope (RI)) or vital dye (usually patent blue). In breast cancer, the combined method concurrently using RI and patent blue has been regarded as the gold standard. Due to the considerable risk of allergic reaction to patent blue [1], the economic costs, and the cosmetically unfavourable blue colouring of the skin, we have been noticing in recent years a steering away from the combined method. In non-complicated cases, some surgeons use only the radioisotope. Meta-analyses have demonstrated that this procedure is safe and does not increase the risk for false negativity of the sentinel biopsy [2, 3]. Even the use of the radioisotope by itself, however, requires that a nuclear medicine facility be available, and this is associated with certain disadvantages relating to radioactivity. Therefore, new methods and indicators are being sought the use of which would be more suitable for clinical practice.

Last year, we published our first experience using superparamagnetic iron oxide particles detected using a magneto-metric probe (SentiMag®) [4]. In accordance with other previously published studies the results of which were summarized in a meta-analysis [5], we did not demonstrate inferiority and reached the conclusions that this method is usable for detecting SLNs in breast cancer and that it demonstrates a similar detection rate and level of false negativity as when a radioisotope is used. However, magnetic detection also is not ideal from the practical perspective. A large volume of the detection substance must be applied (5 ml), plastic instruments must be used in surgery and, above all, a certain amount of the detection substance Sienna+® persists for a long time at the application site. In patients after partial mastectomy, this, according to our own not yet published findings, conditions a brownish skin colouring and creates a marked artefact in magnetic resonance even a year after surgery.

Indocyanine green (ICG) could be another substance potentially usable for detecting SLNs [3, 6]. Approved for medicinal uses in 1956, it is commonly used today for diagnosing liver functions, in ophthalmology, cardiology and microcirculation diagnostics. Intravenous application of ICG to human patients is safe, inasmuch as the substance is quickly excreted from the body in the bile. Upon visual inspection, it has a green colour. In contrast to common dyes, however, it is a fluorophore displaying fluorescence (i.e. the ability to emit a fluorescent signal in incident light). Upon being delivered into the organism, ICG bonds to proteins and other macromolecules. That changes the intensity of fluorescence and the emission spectrum to near infrared (NIR). This radiation is invisible to the human eye but can be detected using special devices with photodynamic cameras [7]. Because the emitted radiation permeates through tissues, in contrast to common dyes, it can also visualize deeply located structures.

A large number of studies directed to use of ICG in detecting SLNs in breast cancer have been published. In a 1999 study by Japanese authors, ICG was used only as a green dye without detecting NIR radiation [8]. With the development of systems allowing for perioperative detection of fluorescence, additional studies used primarily this phenomenon for visualizing lymph vessels and nodes. The first studies from 2008 demonstrating the clinical usability of ICG for visualizing the lymphatic system in the breast [9–16] were followed by studies evaluating primarily the combined use of the dye and ICG [17–21]. In work to optimize the methodology, no clear advantage was demonstrated from applying ICG conjugated with albumin in comparison with application of just ICG [22]. Certain newer studies, including one meta-analysis, addressed the question as to whether detection using ICG is as successful as detection using a radioisotope and whether ICG could be used on its own as an adequate alternative [23–25]. The largest study to date, published by Japanese authors in 2016, tested the use of ICG while detecting fluorescence using the HyperEye system by Mizuho (Japan). Those authors reported a similar SLN detection rate (97.2%) and sensitivity (95.7%) in comparison with the radioisotope. They deduced that detecting SLN in breast cancer using ICG can be a comparable alternative to using the radioisotope [26].

We decided to conduct a pilot study with the objective of verifying the usability of ICG and the HyperEye system for detecting SLNs in breast cancer patients in a European population. The hypothesis was that ICG in combination with the HyperEye system is usable in practice, that the detection rate using ICG reaches values in excess of 90% and that in case of a combined use of ICG and radioisotope, there would be concordance in SLNs displayed with ICG and the radioisotope in an absolute majority of patients. The study was performed at the Masaryk Memorial Cancer Institute (Brno, Czech Republic) during the period February–March 2016 and was approved by the Ethical Committee of the Masaryk Memorial Cancer Institute.

## Methods

The study included 10 female patients with primary breast cancer without clinical signs of axillary lymph node metastases at the time of the diagnosis indicated for partial mastectomy and sentinel lymph node biopsy. To be as close to usual clinical practice as possible, no more stringent entry criteria were established. Conditions for inclusion were provision of informed consent and absence of previously known allergy to indocyanine green or intravenous administration of iodine preparations.

**Fig. 1:**
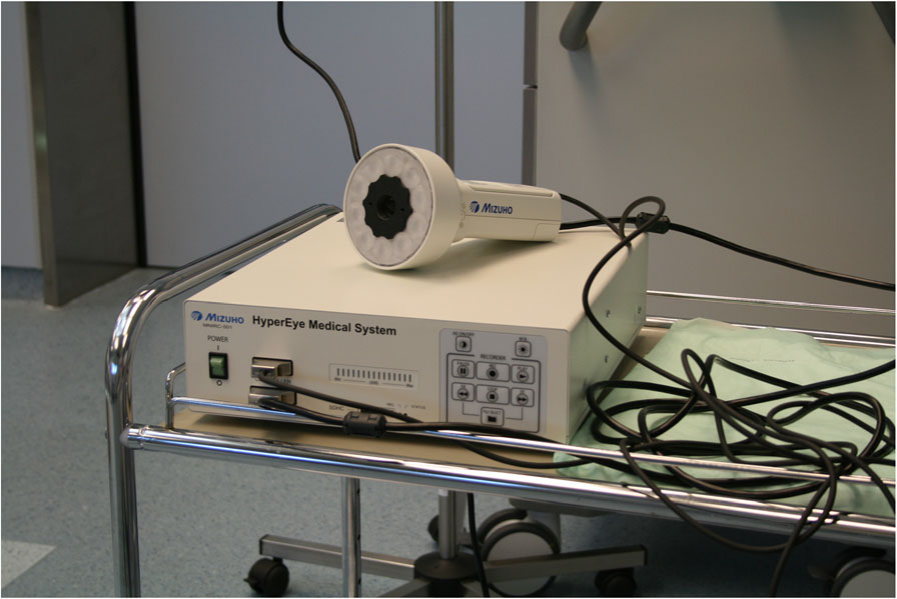
The HyperEye system, Mizuho, Japan

The patients underwent the usual preoperative procedure including ultrasonographic localization of the position of the primary tumour and lymphoscintigraphic examination as part of standard detection of the sentinel node using a radioisotope and a hand-held gamma probe. On surgery day, 99mTc nanocolloid (Nanocoll®) was administered periareolarly in a dose of 100 MBq and the application area was massaged for 20 min. In addition to the usual procedure, ICG was applied after the patient was put under total anaesthesia immediately before the start of surgery. We applied ICG-PULSION 5 mg/ml intradermally and subcutaneously in the periareolar area in the amount of 1 ml of 0.5% solution. This was followed by massaging the application location for at least 1 min. The presence of ICG in the organism was perioperatively detected using the HyperEye system by Mizuho, Japan (Fig. 1), and visualized on a monitor placed on a rotating arm beneath the ceiling of the operating room. The main surgeon was always one of the two authors of this study. Both are surgeons focusing exclusively on breast and skin cancer surgery and with extensive experience in detecting SLNs in a high-throughput work setting. The operating surgeons had been trained in advance on using the HyperEye by an expert representative of the distributor.

In using the HyperEye system, the course of subcutaneous lymphatic vessels was visualized. The surgeon made an incision at the place of the expected location of the SLN. During continuous visualization using a photodynamic camera, he endeavoured to identify and extirpate all nodes demonstrating fluorescence. Radioactivity of the extirpated SLNs was recorded ex vivo using a gamma probe. After this phase of the surgery was completed, the gamma probe was used for checking whether marked substantial peaks of radioactivity persisted in the axilla corresponding to other potential SLNs not identified using fluorescence. If such peaks were discovered, the surgeon looked for and potentially extirpated these additional SLNs. Using the HyperEye system, it was determined ex vivo whether these SLNs manifested fluorescence. The following phase of the surgery was according to usual practice. All extirpated SLNs were examined histologically, and the results were recorded.

The main evaluated parameters were success rate in detecting the SLNs using ICG, match (concordance) in SLNs detected by the individual methods, and with a special focus on the match in detecting SLNs afflicted by metastasis in the follow-up histopathological examination. The numbers of SLNs found using only ICG and using the two methods in combination were compared using Wilcoxon signed-rank test (one-tailed) at the 5% level of significance. Statistical analyses were performed using R version 3.2.4. In addition, the practical aspects as to the usability of ICG and the HyperEye system in clinical practice were assessed.

## Results

**Fig. 2:**
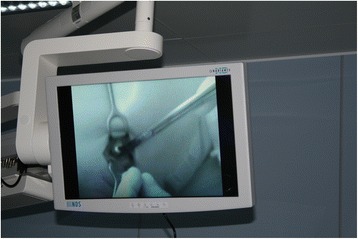
Perioperative visualization of fluorescent lymphatic collector and a sentinel node on a monitor connected to HyperEye

The HyperEye system is usable in practice. Sterility is achieved during surgery by means of a special one-use PVC cover with a rigid plastic transparent camera cover. Before the surgery, it is necessary to turn on the system in advance and to calibrate it. This requires several minutes. It is necessary somewhat to diminish the usual level of lighting, at minimum by temporarily turning the operating lights away from the field of surgery, better by turning off the surrounding lighting or even darkening the room. This is somewhat inconvenient during surgery and extends the operating time. It was found to be impractical to carry out the surgery itself with just the usual surgical team of two surgeons. We brought in a third surgeon to control the photodynamic camera. Figure 2 is a photograph from the operating room demonstrating the visualization on a screen of efferent lymphatic collector and SLN using the HyperEye system.

All the main parameters are apparent from Table 1. In all 10 cases, at least one sentinel node could be detected using ICG itself. The median and mean number of nodes detected and extirpated using ICG was equal to 1 and 1.3, respectively. All such SLNs removed were situated in the typical localization within the ipsilateral axilla and ex vivo demonstrated, in addition to fluorescence, also marked radioactivity. We assume, therefore, that we would have found all these nodes also using RI and the gamma probe. In five cases, no other SLNs were found in subsequent control of the axilla using the gamma probe. In the other five cases, however, between one and four additional SLNs were subsequently found and extirpated using the gamma probe. All these additionally extirpated nodes demonstrated apparent fluorescence ex vivo. Both the median and mean number of SLNs detected per patient using both detection substances therefore reached 2.5. This number of nodes found using both methods was significantly greater than the number of nodes found using only ICG (*p* = 0.03). In one (of 10) case, a macrometastasis was found only in one of the SLNs found subsequently using RI and a gamma probe. Meanwhile, the only SLN detected in that case using ICG was negative. This was, therefore, a case of false negativity of the tested method (ICG) in one case of two where metastatic affliction in SLN occurred (ITC are not considered metastatic afflictions).

**Table 1:** Summary of results

Patient no.	cT	BMI	SLN (ICG)	Additional SLN (RI)	Histology	Note
1	1–2	25.6	1	1	ITC (1/2)	
2	Tis	24.7	1	0		
3	1–2	30.9	3	0		
4	1	27.0	1	0		
5	1	26.0	1	2		
6	1–2	28.9	1	0	Micrometastasis	
7	1	23.1	1	4		
8	1	24.2	1	0		
9	1	25.4	2	1		
10	1	28.7	1	4	Macrometastasis	False negativity of ICG!

Legend: *Patient no*. patient number, *cT* clinical stage of the primary tumour at time of diagnosis, *BMI* body mass index, *SLN (ICG)* number of sentinel lymph nodes detected using ICG, *Additional SLN (RI)* number of sentinel lymph nodes additionally detected using a radioisotope, *Histology* result of histological examination of sentinel lymph nodes, *ITC* isolated tumour cells

## Discussion

Our pilot study did not confirm the hypothesis that detection of SLNs in breast cancer using ICG and the HyperEye system appears to be a fully comparable alternative for detection to using a radioisotope and gamma probe. Using just ICG, we successfully detected at least one SLN in all 10 cases. In that sense, the detection rate was 100%. In half of the cases, however, we subsequently found additional SLNs. The number of SLNs found using both methods was therefore significantly higher than the number of nodes found using only ICG. In one of the patients, a metastasis was found in one of the additionally removed nodes while the only node detected in that patient using ICG was negative. Complete match in SLNs detected using both methods (concordance) was therefore achieved in only 50% of cases. If the level of false negativity of ICG was expressed numerically, it would reach 50%. Considering the small number of cases, however, we do not consider it appropriate to use this number.

All nodes removed using ICG demonstrated ex vivo high values for radioactivity and were localized in the typical place within the ipsilateral axilla. In interpreting the results, we therefore proceed from the assumption that these nodes would have been found also using just RI and gamma probe. There unfortunately is no real way to prove this assumption, however, which is a slight weakness of our study.

There can be several causes of what might be termed the study’s “negative” outcome, which is to say the non-confirmation of the hypothesis as to comparability of ICG and radioisotope methods. There exist various systems for perioperative detection of fluorescence from various manufacturers. The FLARE system has been used for the longest time, and it was relatively bulky and costly in its original form [10]. This was followed by the development of other lines with smaller dimensions [27]. As a practical matter, however, we could not examine these, as was the case also regarding devices from other manufacturers. It is possible that the individual systems differ both in their practical aspects and by their sensitivity in detecting fluorescence radiation. It cannot be excluded, therefore, that we would have reached different results using a different system. In the largest of previously published positive studies [26], however, the same system as in our case was used (HyperEye). It is also probable that the intensity of fluorescence emitted by lymph vessels and nodes can be markedly influenced by the amount and concentration of ICG administered. The specific amount administered differs in the individual studies. In our study, we administered 1 ml of 0.5% solution—similarly as did the authors of the aforementioned robust study—even though the distributor’s representative initially had recommended administration of an even smaller amount (0.5 ml). The published meta-analysis indicates that using a larger volume of the substance in a lower concentration could be more advantageous [24].

All sentinel nodes subsequently extirpated using a gamma probe demonstrated ex vivo apparent fluorescence. The problem is therefore not that ICG would be insufficiently transported into SLNs. Rather, it is apparent that tissues’ permeability to NIR radiation is limited such that the structures can only be visualized to a certain depth not exceeding 1 cm [28]. Certain facts support this notion. After intradermal periareolar application, surface lymph vessels usually converging into a single collector passing over the upper outer quadrant toward the axilla were well visualized on the breast. Several centimetres before the axilla, however, its trace on the skin disappeared, which we explain by the collector’s sinking deeper into the subcutaneous tissue. Fluorescent nodes were therefore not apparent through the skin. It was necessary to perform an incision into the expected location of the SLN and to dissect it. The fluorescence emerging from the efferent lymph vessel or SLN started to be visible only deeper in the subcutaneous tissue or under the axillary fascia. For a similar reason, in eight cases out of 10, we found only one node using ICG. If the other sentinel (fluorescent) nodes were not situated in the immediate vicinity of the first node removed but lay deeper in the undissected tissue, they were not visible. We intentionally avoided larger “blind” dissection in the axilla as we would regard that as excessive damage to the patients. Our assumption as to the crucial influence of depth of the detected structures on the success rate of visualization is supported also by the conclusions of several other studies. German authors have stated that body mass index (BMI) higher than 40 is a limiting factor for using ICG in breast cancer [29]. Although the highest BMI in our patient set reached only 30.9, it is apparent that deeper location of the SLNs in patients with a stronger subcutaneous layer complicates their fluorescent detection. In malignant melanomas, where the approximate location of the sentinel node cannot be empirically so well assumed, the node location could be visualized percutaneously using fluorescence only in 17 cases of 80, and the authors of that study conclusively state that radioisotope remains the gold standard for melanoma [30]. There exists a possibility that a factor contributing to the positive results of studies performed in Asia is the habitus of the local patients, which is somewhat different from that of the European population. Sentinel lymph nodes are located at a shallower depth, so the fluorescent signal can be more apparent on the body surface.

The method cannot be considered ideal even with respect to the practical use of ICG and the HyperEye system. In order to preserve the surgeon’s comfort, an additional worker is needed to control the camera. Moreover, the darkening of the operating room during visualization is somewhat inconvenient. Overall financial costs must include those for depreciation of the device, a special single-use cover with a plastic cover, and the tracer (ICG).

## Conclusions

Our pilot study confirmed a high detection rate of SLNs in breast cancer patients using ICG and the HyperEye system. It did not, however, confirm the assumption that this method could be a fully comparable alternative in a European population of patients to detecting SLNs using radioisotope and gamma probe. This probably was caused by the limited permeability of tissues to fluorescent radiation, which decidedly limits visualization of more deeply situated lymph vessels and nodes.

## Abbreviations

ICG: Indocyanine green
NIR: Near infrared (fluorescence)
RI: Radioisotope
SLN: Sentinel lymph node
